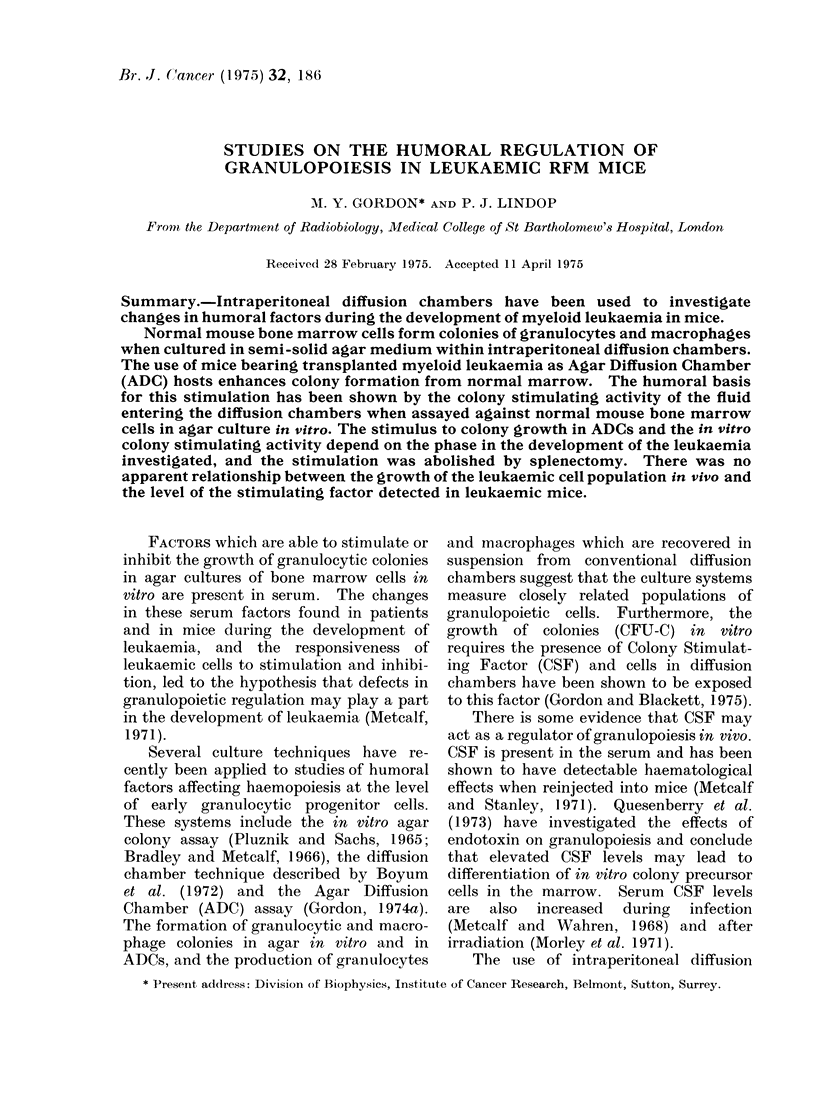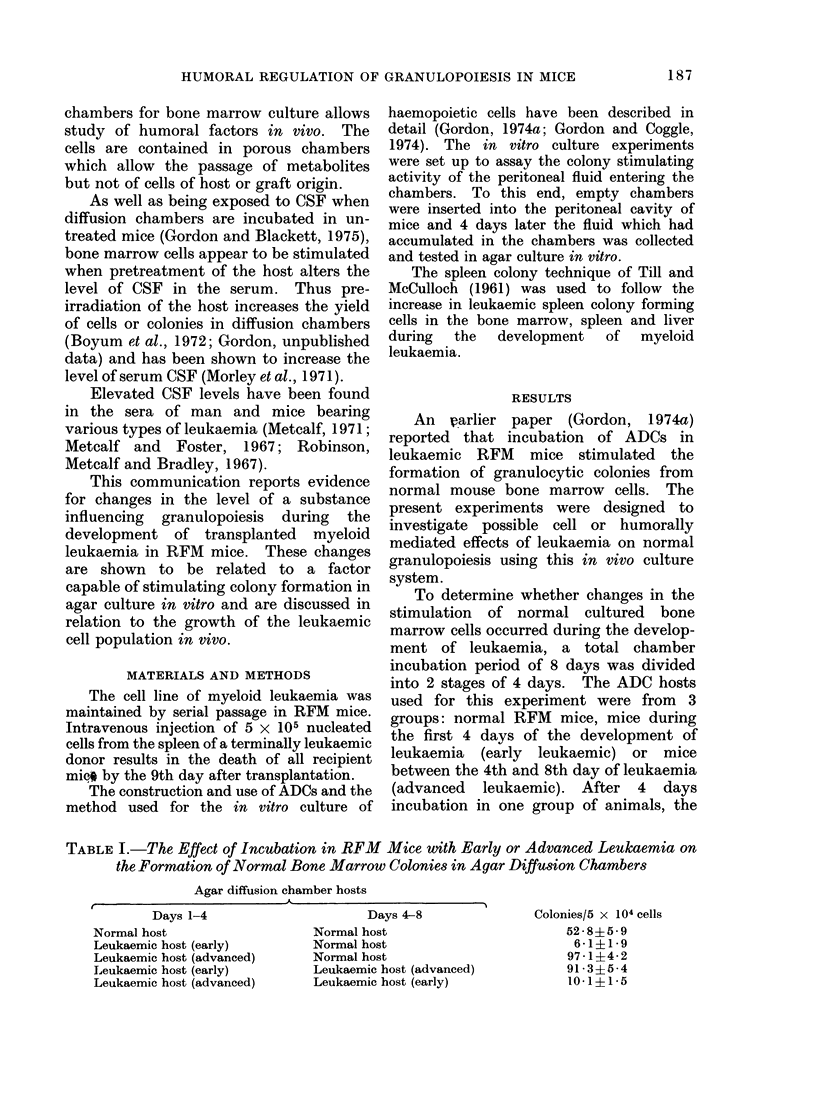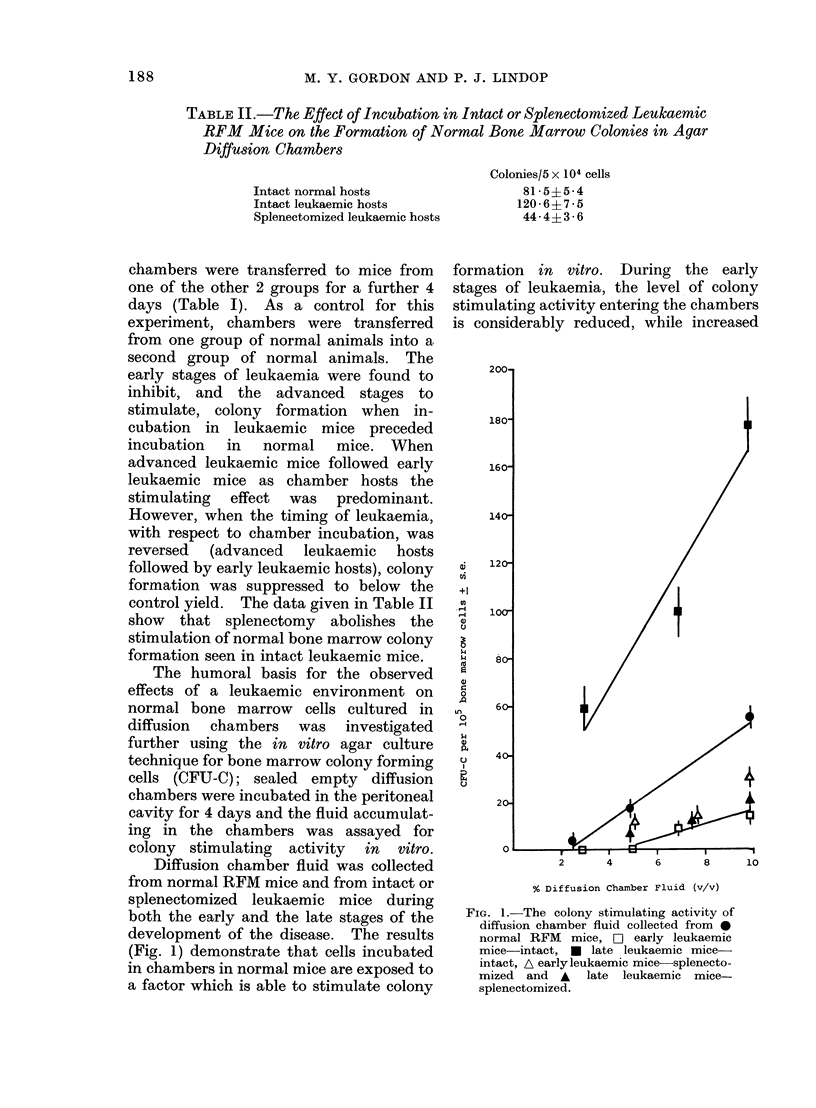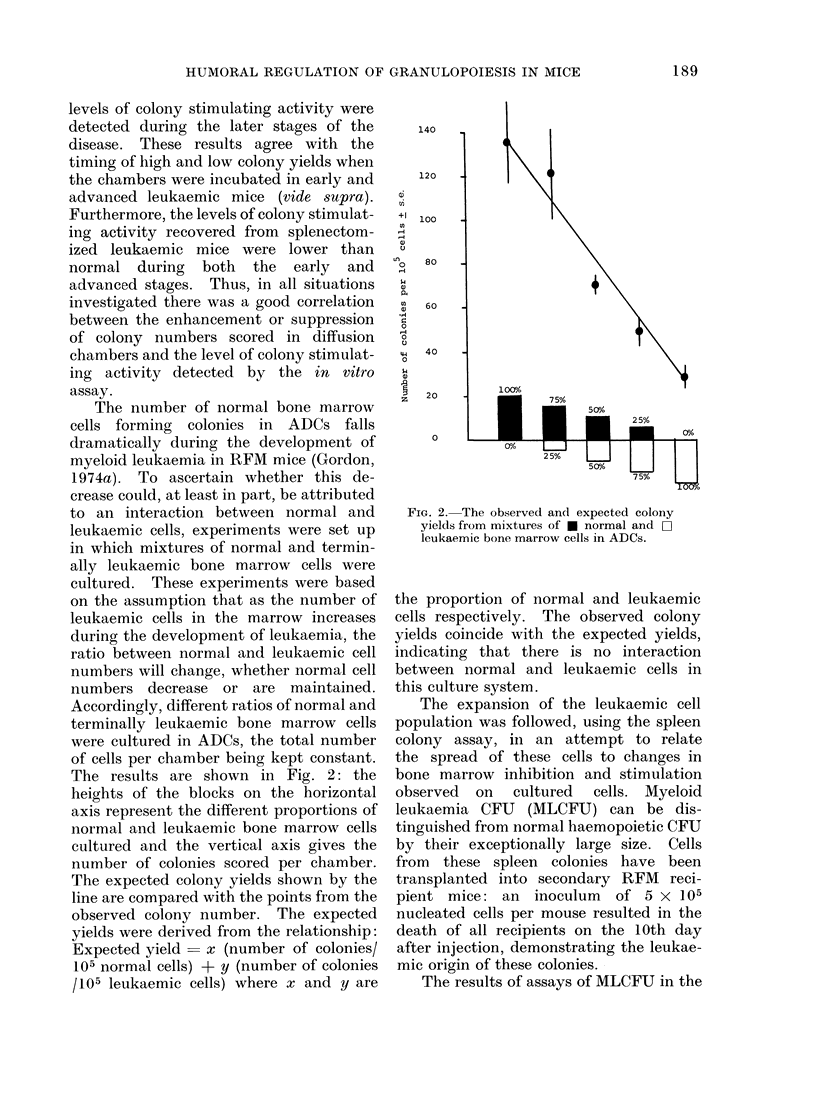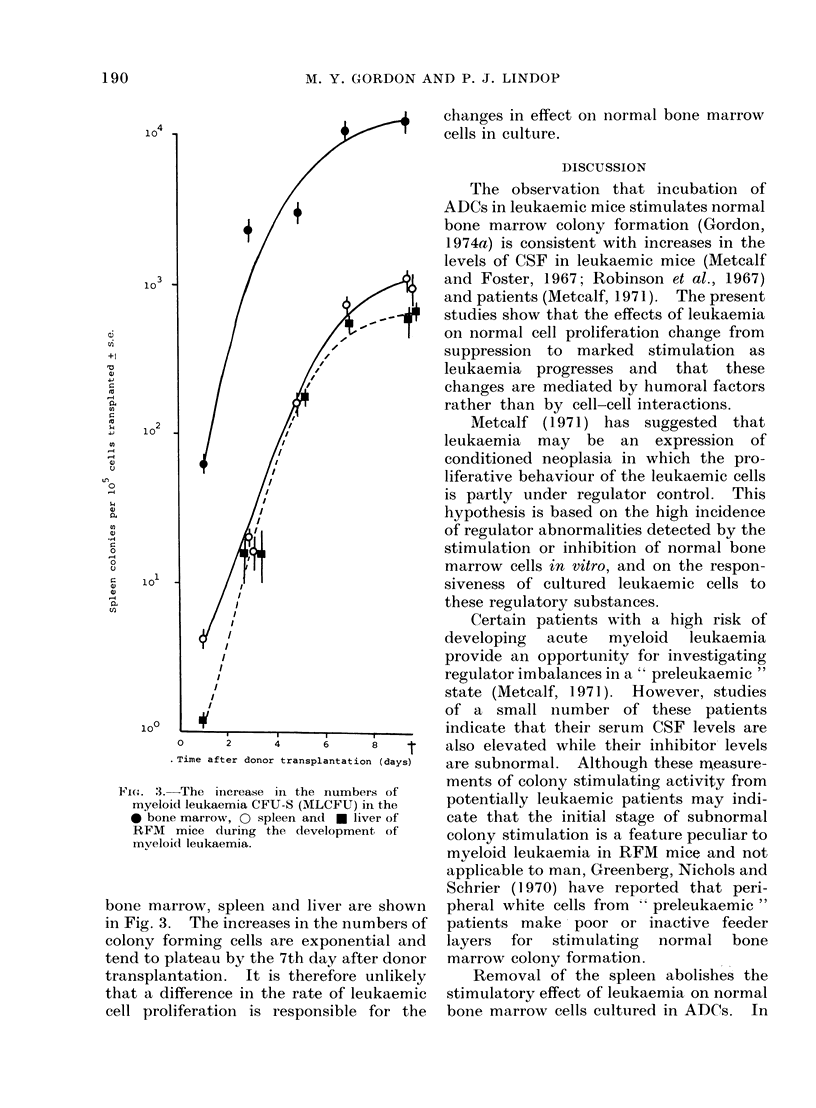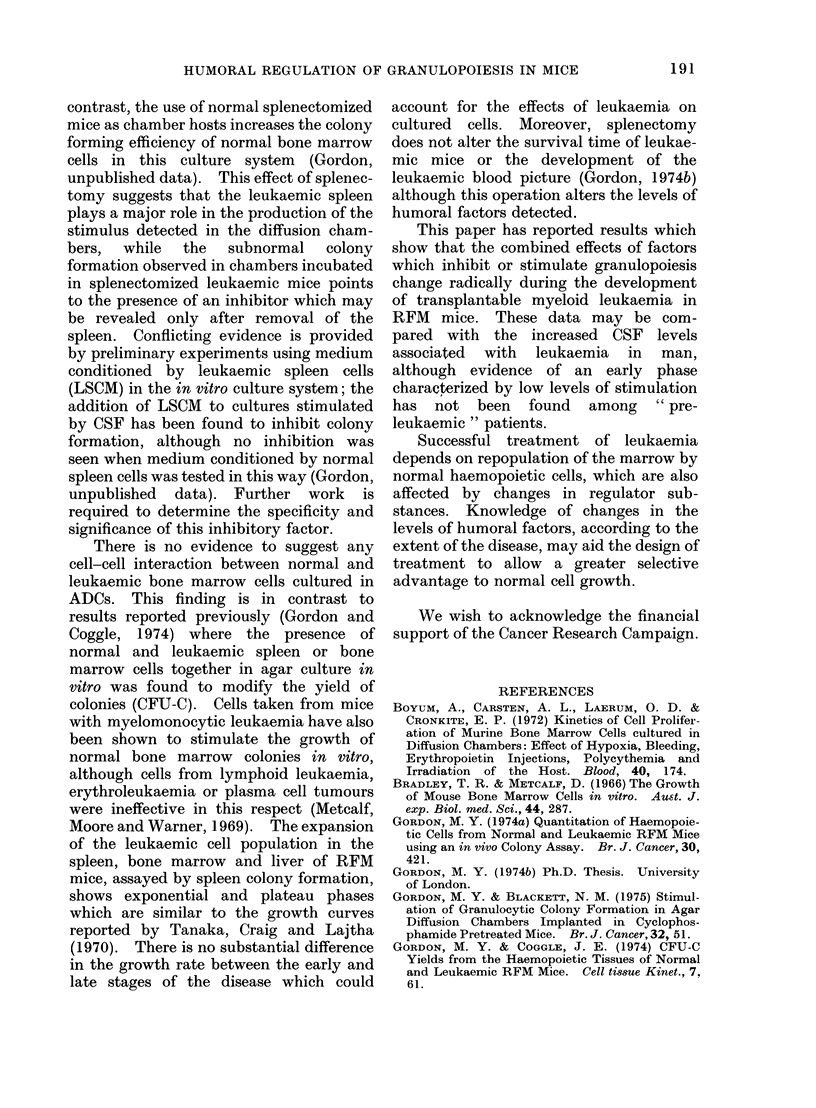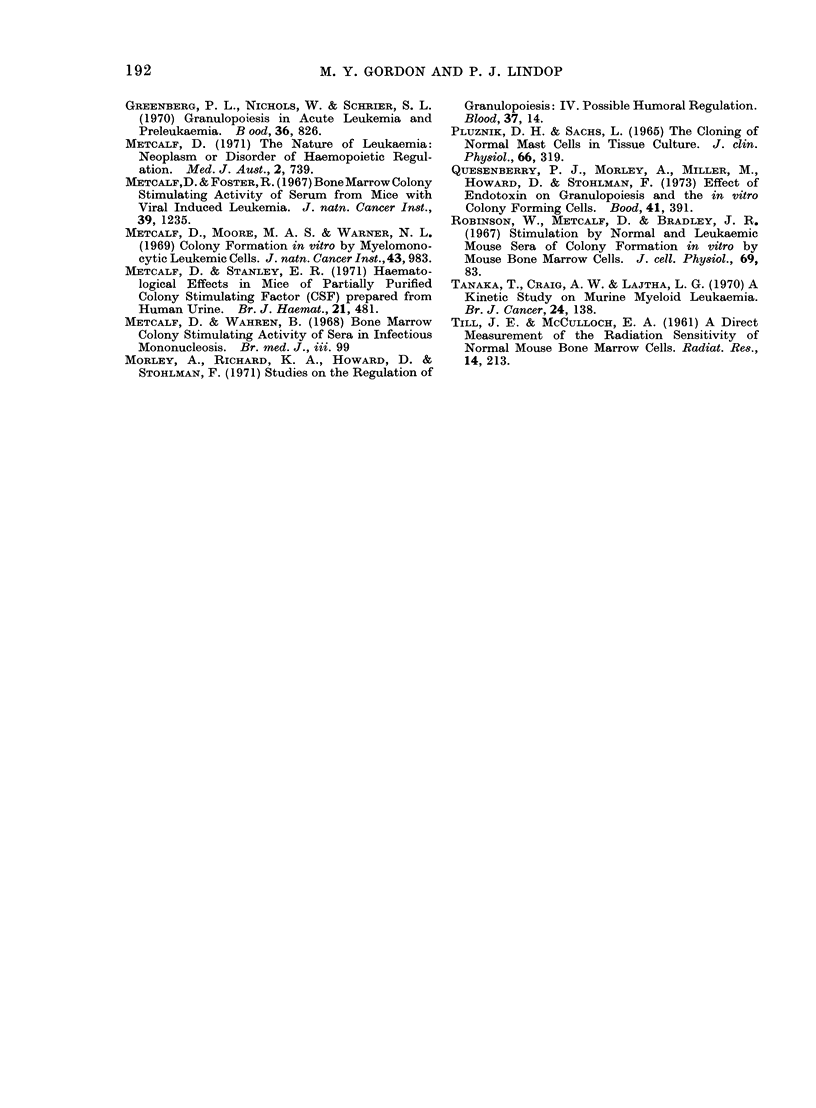# Studies on the humoral regulation of granulopoiesis in leukaemic RFM mice.

**DOI:** 10.1038/bjc.1975.148

**Published:** 1975-08

**Authors:** M. Y. Gordon, P. J. Lindop

## Abstract

Intraperitoneal diffusion chambers have been used to investigate changes in humoral factors during the development of myeloid leukaemia in mice. Normal mouse bone marrow cells form colonies of granulocytes and macrophages when cultured in semi-solid agar medium within intraperitoneal diffusion chambers. The use of mice bearing transplanted myeloid leukaemia as Agar Diffusion Chamber (ADC) hosts enhances colony formation from normal marrow. The humoral basis for this stimulation has been shown by the colony stimulating activity of the fluid entering the diffusion chambers when assayed against normal mouse bone marrow cells in agar culture in vitro. The stimulus to colony growth in ADCs and the in vitro colony stimulating activity depend on the phase in the development of the leukaemia investigated, and the stimulation was abolished by splenectomy. There was no apparent relationship between the growth of the leukaemic cell population in vivo and the level of the stimulating factor detected in leukaemic mice.


*Br. J. Cancer* (1975) **32**, 186

### STUDIES ON THE HUMORAL REGULATION OF GRANULOPOIESIS IN LEUKAEMIC RFM MICE

M. Y. GORDON* AND P. J. LINDOP

*From the Department of Radiobiology, Medical College of St Bartholomew's Hospital, London*

Received 28 February 1975. Accepted 11 April 1975

**Summary.—Intraperitoneal diffusion chambers have been used to investigate changes in humoral factors during the development of myeloid leukaemia in mice.**

**Normal mouse bone marrow cells form colonies of granulocytes and macrophages when cultured in semi-solid agar medium within intraperitoneal diffusion chambers. The use of mice bearing transplanted myeloid leukaemia as Agar Diffusion Chamber (ADC) hosts enhances colony formation from normal marrow. The humoral basis for this stimulation has been shown by the colony stimulating activity of the fluid entering the diffusion chambers when assayed against normal mouse bone marrow cells in agar culture *in vitro*. The stimulus to colony growth in ADCs and the *in vitro* colony stimulating activity depend on the phase in the development of the leukaemia investigated, and the stimulation was abolished by splenectomy. There was no apparent relationship between the growth of the leukaemic cell population *in vivo* and the level of the stimulating factor detected in leukaemic mice.**

FACTORS which are able to stimulate or inhibit the growth of granulocytic colonies in agar cultures of bone marrow cells *in vitro* are present in serum. The changes in these serum factors found in patients and in mice during the development of leukaemia, and the responsiveness of leukaemic cells to stimulation and inhibition, led to the hypothesis that defects in granulopoietic regulation may play a part in the development of leukaemia (Metcalf, 1971).

Several culture techniques have recently been applied to studies of humoral factors affecting haemopoiesis at the level of early granulocytic progenitor cells. These systems include the *in vitro* agar colony assay (Pluznik and Sachs, 1965; Bradley and Metcalf, 1966), the diffusion chamber technique described by Boyum *et al.* (1972) and the Agar Diffusion Chamber (ADC) assay (Gordon, 1974*a*). The formation of granulocytic and macrophage colonies in agar *in vitro* and in ADCs, and the production of granulocytes and macrophages which are recovered in suspension from conventional diffusion chambers suggest that the culture systems measure closely related populations of granulopoietic cells. Furthermore, the growth of colonies (CFU-C) *in vitro* requires the presence of Colony Stimulating Factor (CSF) and cells in diffusion chambers have been shown to be exposed to this factor (Gordon and Blackett, 1975).

There is some evidence that CSF may act as a regulator of granulopoiesis *in vivo*. CSF is present in the serum and has been shown to have detectable haematological effects when reinjected into mice (Metcalf and Stanley, 1971). Quesenberry *et al.* (1973) have investigated the effects of endotoxin on granulopoiesis and conclude that elevated CSF levels may lead to differentiation of *in vitro* colony precursor cells in the marrow. Serum CSF levels are also increased during infection (Metcalf and Wahren, 1968) and after irradiation (Morley *et al.* 1971).

The use of intraperitoneal diffusion

* Present address: Division of Biophysics, Institute of Cancer Research, Belmont, Sutton, Surrey.

chambers for bone marrow culture allows study of humoral factors *in vivo*. The cells are contained in porous chambers which allow the passage of metabolites but not of cells of host or graft origin.

As well as being exposed to CSF when diffusion chambers are incubated in untreated mice (Gordon and Blackett, 1975), bone marrow cells appear to be stimulated when pretreatment of the host alters the level of CSF in the serum. Thus preirradiation of the host increases the yield of cells or colonies in diffusion chambers (Boyum *et al.*, 1972; Gordon, unpublished data) and has been shown to increase the level of serum CSF (Morley *et al.*, 1971).

Elevated CSF levels have been found in the sera of man and mice bearing various types of leukaemia (Metcalf, 1971; Metcalf and Foster, 1967; Robinson, Metcalf and Bradley, 1967).

This communication reports evidence for changes in the level of a substance influencing granulopoiesis during the development of transplanted myeloid leukaemia in RFM mice. These changes are shown to be related to a factor capable of stimulating colony formation in agar culture *in vitro* and are discussed in relation to the growth of the leukaemic cell population *in vivo*.

### MATERIALS AND METHODS

The cell line of myeloid leukaemia was maintained by serial passage in RFM mice. Intravenous injection of $5 \times 10^5$ nucleated cells from the spleen of a terminally leukaemic donor results in the death of all recipient mice by the 9th day after transplantation.

The construction and use of ADCs and the method used for the *in vitro* culture of haemopoietic cells have been described in detail (Gordon, 1974*a*; Gordon and Coggle, 1974). The *in vitro* culture experiments were set up to assay the colony stimulating activity of the peritoneal fluid entering the chambers. To this end, empty chambers were inserted into the peritoneal cavity of mice and 4 days later the fluid which had accumulated in the chambers was collected and tested in agar culture *in vitro*.

The spleen colony technique of Till and McCulloch (1961) was used to follow the increase in leukaemic spleen colony forming cells in the bone marrow, spleen and liver during the development of myeloid leukaemia.

### RESULTS

An earlier paper (Gordon, 1974*a*) reported that incubation of ADCs in leukaemic RFM mice stimulated the formation of granulocytic colonies from normal mouse bone marrow cells. The present experiments were designed to investigate possible cell or humorally mediated effects of leukaemia on normal granulopoiesis using this *in vivo* culture system.

To determine whether changes in the stimulation of normal cultured bone marrow cells occurred during the development of leukaemia, a total chamber incubation period of 8 days was divided into 2 stages of 4 days. The ADC hosts used for this experiment were from 3 groups: normal RFM mice, mice during the first 4 days of the development of leukaemia (early leukaemic) or mice between the 4th and 8th day of leukaemia (advanced leukaemic). After 4 days incubation in one group of animals, the

TABLE I.—*The Effect of Incubation in RFM Mice with Early or Advanced Leukaemia on the Formation of Normal Bone Marrow Colonies in Agar Diffusion Chambers*

| Agar diffusion chamber hosts | | |
|---|---|---|
| Days 1–4 | Days 4–8 | Colonies/$5 \times 10^4$ cells |
| Normal host | Normal host | $52 \cdot 8 \pm 5 \cdot 9$ |
| Leukaemic host (early) | Normal host | $6 \cdot 1 \pm 1 \cdot 9$ |
| Leukaemic host (advanced) | Normal host | $97 \cdot 1 \pm 4 \cdot 2$ |
| Leukaemic host (early) | Leukaemic host (advanced) | $91 \cdot 3 \pm 5 \cdot 4$ |
| Leukaemic host (advanced) | Leukaemic host (early) | $10 \cdot 1 \pm 1 \cdot 5$ |

TABLE II.—*The Effect of Incubation in Intact or Splenectomized Leukaemic RFM Mice on the Formation of Normal Bone Marrow Colonies in Agar Diffusion Chambers*

| | Colonies/$5 \times 10^4$ cells |
|---|---|
| Intact normal hosts | $81 \cdot 5 \pm 5 \cdot 4$ |
| Intact leukaemic hosts | $120 \cdot 6 \pm 7 \cdot 5$ |
| Splenectomized leukaemic hosts | $44 \cdot 4 \pm 3 \cdot 6$ |

chambers were transferred to mice from one of the other 2 groups for a further 4 days (Table I). As a control for this experiment, chambers were transferred from one group of normal animals into a second group of normal animals. The early stages of leukaemia were found to inhibit, and the advanced stages to stimulate, colony formation when incubation in leukaemic mice preceded incubation in normal mice. When advanced leukaemic mice followed early leukaemic mice as chamber hosts the stimulating effect was predominant. However, when the timing of leukaemia, with respect to chamber incubation, was reversed (advanced leukaemic hosts followed by early leukaemic hosts), colony formation was suppressed to below the control yield. The data given in Table II show that splenectomy abolishes the stimulation of normal bone marrow colony formation seen in intact leukaemic mice.

The humoral basis for the observed effects of a leukaemic environment on normal bone marrow cells cultured in diffusion chambers was investigated further using the *in vitro* agar culture technique for bone marrow colony forming cells (CFU-C); sealed empty diffusion chambers were incubated in the peritoneal cavity for 4 days and the fluid accumulating in the chambers was assayed for colony stimulating activity *in vitro*.

Diffusion chamber fluid was collected from normal RFM mice and from intact or splenectomized leukaemic mice during both the early and the late stages of the development of the disease. The results (Fig. 1) demonstrate that cells incubated in chambers in normal mice are exposed to a factor which is able to stimulate colony formation *in vitro*. During the early stages of leukaemia, the level of colony stimulating activity entering the chambers is considerably reduced, while increased

FIG. 1.—The colony stimulating activity of diffusion chamber fluid collected from ● normal RFM mice, □ early leukaemic mice—intact, ■ late leukaemic mice—intact, △ early leukaemic mice—splenectomized and ▲ late leukaemic mice—splenectomized.

levels of colony stimulating activity were detected during the later stages of the disease. These results agree with the timing of high and low colony yields when the chambers were incubated in early and advanced leukaemic mice (*vide supra*). Furthermore, the levels of colony stimulating activity recovered from splenectomized leukaemic mice were lower than normal during both the early and advanced stages. Thus, in all situations investigated there was a good correlation between the enhancement or suppression of colony numbers scored in diffusion chambers and the level of colony stimulating activity detected by the *in vitro* assay.

The number of normal bone marrow cells forming colonies in ADCs falls dramatically during the development of myeloid leukaemia in RFM mice (Gordon, 1974*a*). To ascertain whether this decrease could, at least in part, be attributed to an interaction between normal and leukaemic cells, experiments were set up in which mixtures of normal and terminally leukaemic bone marrow cells were cultured. These experiments were based on the assumption that as the number of leukaemic cells in the marrow increases during the development of leukaemia, the ratio between normal and leukaemic cell numbers will change, whether normal cell numbers decrease or are maintained. Accordingly, different ratios of normal and terminally leukaemic bone marrow cells were cultured in ADCs, the total number of cells per chamber being kept constant. The results are shown in Fig. 2: the heights of the blocks on the horizontal axis represent the different proportions of normal and leukaemic bone marrow cells cultured and the vertical axis gives the number of colonies scored per chamber. The expected colony yields shown by the line are compared with the points from the observed colony number. The expected yields were derived from the relationship: Expected yield $= x$ (number of colonies/$10^5$ normal cells) $+ y$ (number of colonies /$10^5$ leukaemic cells) where $x$ and $y$ are the proportion of normal and leukaemic cells respectively. The observed colony yields coincide with the expected yields, indicating that there is no interaction between normal and leukaemic cells in this culture system.

FIG. 2.—The observed and expected colony yields from mixtures of ■ normal and □ leukaemic bone marrow cells in ADCs.

The expansion of the leukaemic cell population was followed, using the spleen colony assay, in an attempt to relate the spread of these cells to changes in bone marrow inhibition and stimulation observed on cultured cells. Myeloid leukaemia CFU (MLCFU) can be distinguished from normal haemopoietic CFU by their exceptionally large size. Cells from these spleen colonies have been transplanted into secondary RFM recipient mice: an inoculum of $5 \times 10^5$ nucleated cells per mouse resulted in the death of all recipients on the 10th day after injection, demonstrating the leukaemic origin of these colonies.

The results of assays of MLCFU in the

FIG. 3.—The increase in the numbers of myeloid leukaemia CFU-S (MLCFU) in the ● bone marrow, ○ spleen and ■ liver of RFM mice during the development of myeloid leukaemia.

bone marrow, spleen and liver are shown in Fig. 3. The increases in the numbers of colony forming cells are exponential and tend to plateau by the 7th day after donor transplantation. It is therefore unlikely that a difference in the rate of leukaemic cell proliferation is responsible for the changes in effect on normal bone marrow cells in culture.

### DISCUSSION

The observation that incubation of ADCs in leukaemic mice stimulates normal bone marrow colony formation (Gordon, 1974*a*) is consistent with increases in the levels of CSF in leukaemic mice (Metcalf and Foster, 1967; Robinson *et al.*, 1967) and patients (Metcalf, 1971). The present studies show that the effects of leukaemia on normal cell proliferation change from suppression to marked stimulation as leukaemia progresses and that these changes are mediated by humoral factors rather than by cell–cell interactions.

Metcalf (1971) has suggested that leukaemia may be an expression of conditioned neoplasia in which the proliferative behaviour of the leukaemic cells is partly under regulator control. This hypothesis is based on the high incidence of regulator abnormalities detected by the stimulation or inhibition of normal bone marrow cells *in vitro*, and on the responsiveness of cultured leukaemic cells to these regulatory substances.

Certain patients with a high risk of developing acute myeloid leukaemia provide an opportunity for investigating regulator imbalances in a " preleukaemic " state (Metcalf, 1971). However, studies of a small number of these patients indicate that their serum CSF levels are also elevated while their inhibitor levels are subnormal. Although these measurements of colony stimulating activity from potentially leukaemic patients may indicate that the initial stage of subnormal colony stimulation is a feature peculiar to myeloid leukaemia in RFM mice and not applicable to man, Greenberg, Nichols and Schrier (1970) have reported that peripheral white cells from " preleukaemic " patients make poor or inactive feeder layers for stimulating normal bone marrow colony formation.

Removal of the spleen abolishes the stimulatory effect of leukaemia on normal bone marrow cells cultured in ADCs. In contrast, the use of normal splenectomized mice as chamber hosts increases the colony forming efficiency of normal bone marrow cells in this culture system (Gordon, unpublished data). This effect of splenectomy suggests that the leukaemic spleen plays a major role in the production of the stimulus detected in the diffusion chambers, while the subnormal colony formation observed in chambers incubated in splenectomized leukaemic mice points to the presence of an inhibitor which may be revealed only after removal of the spleen. Conflicting evidence is provided by preliminary experiments using medium conditioned by leukaemic spleen cells (LSCM) in the *in vitro* culture system; the addition of LSCM to cultures stimulated by CSF has been found to inhibit colony formation, although no inhibition was seen when medium conditioned by normal spleen cells was tested in this way (Gordon, unpublished data). Further work is required to determine the specificity and significance of this inhibitory factor.

There is no evidence to suggest any cell–cell interaction between normal and leukaemic bone marrow cells cultured in ADCs. This finding is in contrast to results reported previously (Gordon and Coggle, 1974) where the presence of normal and leukaemic spleen or bone marrow cells together in agar culture *in vitro* was found to modify the yield of colonies (CFU-C). Cells taken from mice with myelomonocytic leukaemia have also been shown to stimulate the growth of normal bone marrow colonies *in vitro*, although cells from lymphoid leukaemia, erythroleukaemia or plasma cell tumours were ineffective in this respect (Metcalf, Moore and Warner, 1969). The expansion of the leukaemic cell population in the spleen, bone marrow and liver of RFM mice, assayed by spleen colony formation, shows exponential and plateau phases which are similar to the growth curves reported by Tanaka, Craig and Lajtha (1970). There is no substantial difference in the growth rate between the early and late stages of the disease which could account for the effects of leukaemia on cultured cells. Moreover, splenectomy does not alter the survival time of leukaemic mice or the development of the leukaemic blood picture (Gordon, 1974*b*) although this operation alters the levels of humoral factors detected.

This paper has reported results which show that the combined effects of factors which inhibit or stimulate granulopoiesis change radically during the development of transplantable myeloid leukaemia in RFM mice. These data may be compared with the increased CSF levels associated with leukaemia in man, although evidence of an early phase characterized by low levels of stimulation has not been found among "pre-leukaemic" patients.

Successful treatment of leukaemia depends on repopulation of the marrow by normal haemopoietic cells, which are also affected by changes in regulator substances. Knowledge of changes in the levels of humoral factors, according to the extent of the disease, may aid the design of treatment to allow a greater selective advantage to normal cell growth.

We wish to acknowledge the financial support of the Cancer Research Campaign.

### REFERENCES

BOYUM, A., CARSTEN, A. L., LAERUM, O. D. & CRONKITE, E. P. (1972) Kinetics of Cell Proliferation of Murine Bone Marrow Cells cultured in Diffusion Chambers: Effect of Hypoxia, Bleeding, Erythropoietin Injections, Polycythemia and Irradiation of the Host. *Blood*, **40**, 174.

BRADLEY, T. R. & METCALF, D. (1966) The Growth of Mouse Bone Marrow Cells *in vitro*. *Aust. J. exp. Biol. med. Sci.*, **44**, 287.

GORDON, M. Y. (1974*a*) Quantitation of Haemopoietic Cells from Normal and Leukaemic RFM Mice using an *in vivo* Colony Assay. *Br. J. Cancer*, **30**, 421.

GORDON, M. Y. (1974*b*) Ph.D. Thesis. University of London.

GORDON, M. Y. & BLACKETT, N. M. (1975) Stimulation of Granulocytic Colony Formation in Agar Diffusion Chambers Implanted in Cyclophosphamide Pretreated Mice. *Br. J. Cancer*, **32**, 51.

GORDON, M. Y. & COGGLE, J. E. (1974) CFU-C Yields from the Haemopoietic Tissues of Normal and Leukaemic RFM Mice. *Cell tissue Kinet.*, **7**, 61.

GREENBERG, P. L., NICHOLS, W. & SCHRIER, S. L. (1970) Granulopoiesis in Acute Leukemia and Preleukaemia. *B ood*, **36**, 826.

METCALF, D. (1971) The Nature of Leukaemia: Neoplasm or Disorder of Haemopoietic Regulation. *Med. J. Aust.*, **2**, 739.

METCALF, D. & FOSTER, R. (1967) Bone Marrow Colony Stimulating Activity of Serum from Mice with Viral Induced Leukemia. *J. natn. Cancer Inst.*, **39**, 1235.

METCALF, D., MOORE, M. A. S. & WARNER, N. L. (1969) Colony Formation *in vitro* by Myelomonocytic Leukemic Cells. *J. natn. Cancer Inst.*, **43**, 983.

METCALF, D. & STANLEY, E. R. (1971) Haematological Effects in Mice of Partially Purified Colony Stimulating Factor (CSF) prepared from Human Urine. *Br. J. Haemat.*, **21**, 481.

METCALF, D. & WAHREN, B. (1968) Bone Marrow Colony Stimulating Activity of Sera in Infectious Mononucleosis. *Br. med. J.*, *iii*. 99

MORLEY, A., RICHARD, K. A., HOWARD, D. & STOHLMAN, F. (1971) Studies on the Regulation of Granulopoiesis: IV. Possible Humoral Regulation. *Blood*, **37**, 14.

PLUZNIK, D. H. & SACHS, L. (1965) The Cloning of Normal Mast Cells in Tissue Culture. *J. clin. Physiol.*, **66**, 319.

QUESENBERRY, P. J., MORLEY, A., MILLER, M., HOWARD, D. & STOHLMAN, F. (1973) Effect of Endotoxin on Granulopoiesis and the *in vitro* Colony Forming Cells. *Bood*, **41**, 391.

ROBINSON, W., METCALF, D. & BRADLEY, J. R. (1967) Stimulation by Normal and Leukaemic Mouse Sera of Colony Formation *in vitro* by Mouse Bone Marrow Cells. *J. cell. Physiol.*, **69**, 83.

TANAKA, T., CRAIG, A. W. & LAJTHA, L. G. (1970) A Kinetic Study on Murine Myeloid Leukaemia. *Br. J. Cancer*, **24**, 138.

TILL, J. E. & MCCULLOCH, E. A. (1961) A Direct Measurement of the Radiation Sensitivity of Normal Mouse Bone Marrow Cells. *Radiat. Res.*, **14**, 213.